# Pattern of risky sexual behavior and associated factors among undergraduate students of the University of Port Harcourt, Rivers State, Nigeria

**Published:** 2012-08-07

**Authors:** John Abdulrahman Imaledo, Opirite Boma Peter-Kio, Eme Olukemi Asuquo

**Affiliations:** 1 Department of Health Promotion and Education, University of Ibadan, Nigeria; 2 Department of Human Kinetics, Health and Safety Education, Rivers State University of Education, Nigeria; 3 Department of Preventive and Social Medicine, University of Port-Harcourt, Nigeria

**Keywords:** isky sexual behaviour, pattern, undergraduate

## Abstract

**Introduction:**

Age at sexual debut had decreased in the developing countries recently. Few documented studies have looked into the pattern of risky sexual behaviour and associated factors among undergraduate students in Rivers state of recent. This study examined the pattern of sexual risky behaviour of undergraduate in University of Port-Harcourt, Rivers State, Nigeria.

**Methods:**

A descriptive cross sectional survey was adopted and three hundred students were purposively recruited. Data were collected by a self –administered semi-structured questionnaire and analysed using descriptive and chi-square statistics at 0.05 significant level.

**Results:**

The mean age of respondents was 21.3±2.8years; more than half (57.4%) were females. Almost a quarter (24.5%) was each in 200 and 300 level of study respectively and more than a quarter (26.7%) lives alone. Sixty-one percent of the respondents had ever taken alcohol beverage with 36.1% of them were current users. More than half (52.0%) the respondents had either boy/girlfriend and a total of 144 (52.0%) had ever had sexual intercourse; of these 13% reportedly had only one sexual partner in their lifetime; girl/boy friend topped the list of their sexual partner; 48.6% respondents were currently sexually active and 32% used a form of protection during their last sexual activity. The mean age at sexual debut was 17.0± 4.5years. Few (13.4%) have had sex in exchange for gifts and 5.1% of these was with a friend. Higher proportions of respondents who reported lifetime use of alcohol were sexually active (p<0.005).

**Conclusion:**

Respondents exhibits risky sexual behavior as more had sex without any form of protection. Public enlightenment programmes and targeted behavioral change interventions are therefore recommended.

## Background

There are about 2 billion people between 10-24 years old in the world, close to 85% of these young men and women live in developing countries [1]. The youths in Nigeria account for 32.0% of Nigerian's 140 million people and nearly half (48.6%) of adolescents aged 15-19 are sexually active [2]. About 1 in 5 of sexually active females and 1 in 12 sexually active males had already engaged in sexual intercourse by the age of 15. Findings from National AIDS and Reproductive Health Survey show that the median age of sexual debut among youths is 17years in females and 21years in males. A common feature of young people in Nigeria is their potential vulnerability to Sexually Transmitted Infections (STI) including HIV [2].

Physiologically, the changes in reproductive organs that occur in the life of adolescents often serve as a motivating force in their quest to experiment with sex. Some naturally explore and take risks in many aspects of their lives, including sexual relationships. Those who have sex may change partners frequently and have more than one partner in the same time period or engage in unprotected sex. These risky sexual activities make this group disproportionately affected by reproductive morbidities including STI/HIV, unwanted pregnancies and their complications [3–7]. In Nigeria, researches have confirmed that risky sexual behavior is associated with young people. These risky behaviors include: early debut in sexual activities, sex with many partners, low and inconsistent use of condoms, use of drugs and alcohol, anal sexual intercourse and mouth to genital contact [8–12]. It is of concern that many of these young people do not perceive their high- risk status in spite of indulging in these unsafe sexual practices [12]. It is therefore no surprise that the Joint United Nations Programme on AIDS (UNAIDS) reported that the rate of newly acquired HIV infections are the highest in the 15-25 years age- group and that this group accounts for about 60.0% of the global total of HIV infected persons [13]. Similarly, the highest sero- prevalence rate of HIV in Nigeria is in this age-group [14].

The majority of students in tertiary institutions are single, young adults who easily fall prey to exuberance coupled with the liberal nature of campus life that predisposes them to high risk sexual behaviour. Concerns regarding the implications of this behaviour have led to increasing interventions particularly for in-school adolescents. Though many studies have been carried out to determine the sexual behaviour of adolescents in Nigeria, most of these studies are conducted in the southwest region of the country and few documented studies have looked at the factors that encourage risky sexual behaviour among university students in the south-south region of Nigeria. This study therefore focused on the pattern of risky sexual behaviour of the undergraduate students of the University of Port Harcourt and its associated factors.

## Methods

### Study Design

The study was a descriptive cross-sectional design that explored the pattern of risky sexual behavior and associated factors among undergraduate students of the University of Port Harcourt, in the South-South geo-political zone of Nigeria.

### Method of data collection

The quantitative method of data collection was adopted for this study to gain insights into the context in which risky sexual behaviour are been practiced among university students and make suggestions for feasible interventions in this population.

### Study site

The study population was undergraduate students of the University of Port Harcourt. As at 2010/2011 academic session, when the study was conducted, the university had students’ population of over 20,000. The University of Port Harcourt was established by the Federal Military Government in 1975 as a University College. The University started its first academic session in October 1977 with degree programmes offered in the following schools: School of Humanities; Social Sciences; Biological Sciences; Chemical Sciences; Physical Sciences and Educational Studies but in 1982/83 session the University changed from the School to the Faculty System and at present has ten (10) faculties namely: Faculty of Humanities; Faculty of Social Sciences; Faculty of Education; Faculty of Engineering; Faculty of Management Sciences; College of Health Sciences; Faculty of Science; Faculty of Dentistry; Faculty of Pharmacy and Faculty of Agriculture.

### Sampling Technique

A non probability sampling (purposive sampling) technique was used to recruit 300 respondents for the study. Purposive sampling technique was used because of the ongoing University examinations at the time of the study and the main criterion for inclusion in the study was that a respondent is an undergraduate student of the University.

### Ethical consideration

The nature, purpose and process of the study were explained to the respondents after which verbal consent were obtained from those who agreed to participate in the study. Respondents were assured of confidentiality, privacy and anonymity of information provided and giving the choice not to partake in the study if they so desired and as many that agreed were recruited for the study.

### Data collection, management and analysis

The questionnaire employed both open-ended and closed-ended questions; it was designed to be self-administered. The questionnaire was divided into two (2) sections. The first section asked for the socio-demographic data of the respondents. The second section assessed the pattern of sexual behavior among undergraduate students of the university. In the context of this paper, risky sexual behaviours that were accessed includes: the number of sexual partners; ever had sex, use of protection during the last sexual episode; use of alcohol and sex in exchange for gifts. The copies of the questionnaire were administered over a period of one week. A total of 300 questionnaires was administered and 277 (92.3%) of these questionnaires were retrieved. The completed questionnaires were checked for completeness and a coding guide was developed to facilitate data entry. The data was analyzed with Statistical Package for Social Sciences (SPSS) software package, version 15.0 using descriptive statistics and chi-square at 0.05 significant level.

## Results

### Social-demographic Characteristics

Majority (66.1%) of respondents were between 0-24 years age and 23.8% between 15-19 years, with a mean age of 21.3±28years. Virtually all (97.8%) the respondents were single, 57.4% were females, 53.1%were from the South-south region of the country and 96.8% were Christians. There were more respondents (24.5%) in the 200 and 300 levels of study than other levels. The faculty affiliation of the students showed that 27.9% were in the faculties of Health sciences, 27.2% in Engineering, 19.6% in Humanities, 16.6% in Sciences and 8.7% in Social Sciences. More than half (57.4%) of the respondents live with their parents while 26.7% of them lived alone. More (64.3%) respondents’ father had tertiary education while 5.2% had mothers with tertiary education (Table 1).

**Table 1:** Socio-demographic characteristics of respondents

Variables	Frequency	Percentage
**Gender (N = 277)**		
Males	118	42.6
females	159	57.4
**Age (in years) (N = 277)**		
15-19	66	23.8
20-24	188	66.1
≥25	28	10.2
**Marital status**		
Married	3	1.1
Single	271	97.8
Co-habiting	3	1.1
**Ethnic origin**		
South-south	147	53.1
South - east	93	33.6
South – West	20	7.2
**Levels of study**		
100	56	20.2
200	68	24.5
300	68	24.5
400	53	19.1
500	26	9.4
600	4	1.4
**Religion (N = 277)**		
Christianity	268	96.8
Islam	7	2.5
Traditional	1	.4
Eckanka	1	.4
**Who do you live with? (N = 277)**		
Parents	159	57.4
family relations	13	4.7
Alone	74	26.7
Boyfriend/girl friend	23	8.3
Guardian	5	1.8
Grand parents	1	.4
Husband	1	.4
**What is your father's level of education (n = 269)**		
No formal education	4	1.4
Primary school	10	3.6
Secondary school	31	11.2
Grade11/technical	36	13.0
Tertiary	178	64.3
Don't know	10	3.6
**What is your mother's level of education (n = 265)**		
No formal education	12	4.3
Primary school	16	5.8
Secondary school	41	14.8
Grade11/technical	32	11.6
Tertiary	153	55.2
Don't know	11	4.0

### Respondents’ at risk behavior

Sixty- one percent of the respondents had taken alcohol with 36.1% of them been current users and only 8.7% are current users of cigarette. More than a quarter (31.4%) and 22.0% of the respondents had fathers who drank alcohol and both parents that drank alcohol respectively (Table 2).

**Table 2:** Respondents’ at risk behaviours

Variables	Frequency	Percentage
**Have you ever taken alcohol (N = 277)**		
Yes	169	66.1
No	91	32.9
No Response	17	6.1
**Do you still drink alcoholic beverages? (N = 277)**		
Yes	100	36.1
No	79	28.5
No Response	98	35.4
**Do you smoke cigarettes? (N = 277)**		
Yes	24	8.7
No	238	85.9
No Response	15	5.4
**Have you ever worked for money?**		
Yes	181	65.3
No	72	26.0
No Response	24	8.7
**Do you work for money now?**		
Yes	42	15.2
No	141	50.9
No Response	94	33.9
**Which of your parents drink alcoholic beverages**		
Father	87	31.4
Mother	7	2.5
Both of them	61	22.0
No response	122	44.0

### Respondents sexual behaviour

More than half of the respondents (52%) had either boyfriend or girlfriend and 144 (52.0%) of the respondents have ever had sex with someone. Age at first sexual intercourse revealed that 33.6% of the respondents had their first sexual intercourse within the age range of 5-19 years (3.2% 5-9years, 5.1% 10-14years, 25.3% 15-19 years)(Table 3), 14.1% 20-24years and 52.3% 24years In answering questions on when last respondents had sex with someone, the results showed that more (30.3%) of them had sexual intercourse in 2011; 23.5% had had sex with someone in the month preceding the study and 13.4% reportedly had one sexual partner. Girl or boy friend topped the list of persons respondents had sex with and only 31.8% of them used a form of protection. Few of the respondents (13.4%) had had sex in exchange for gifts and 5.1% said that the person they exchanged sex for gifts with was a friend. A relationship was established between current user of alcohol and having sex (p<0.05) (Table 4).

**Table 3:** Alcohol usage by sexual intercourse

	Have you had sex		
Do you still drink alcoholic beverages?	Yes	No	Total
Yes	73(77.7%)	21(22.3%)	94(100%)
No	39 (51.3%)	37(48.7%)	76(100%)
Total	112(65.9%)	58(34.1%)	170100%)

χ^2^ = 12.975; df= 1; P=.000

**Table 4:** Alcohol usage by sexual intercourse

Alcohol Usage	Have you had sex with someone		
Do you still drink alcohol beverage	Yes	No	Total
Yes	73(77.7%)	21(22.3%)	94(100%)
No	39 (51.3%)	37(48.7%)	76(100%)
Total	112(65.9%)	58(34.1%)	170100%)

χ^2^ = 12.975; df= 1; P=.000

## Discussion

### Socio-demographic characteristics of respondents

Majority of the respondents were between 15 and 24 years and with a mean age of 21.3±2.8years and most of them were single. This is a reflection that they were relatively young and some were sexually active, this is confirmed by the National Demography Survey Data (NDHS) which revealed that nearly half (48.6%) of adolescents aged 15-19 are sexually active (NDHS, 2008) [2]. In Nigeria and all over the world, the long years of continued education has created a big gap between the age of puberty and age at marriage, thus increasing the likelihood of sexual initiation and unprotected premarital sex, it thus create a situation where people are students and at the same time sexually active.

In this study, females were more than males. These is due to the fact that at the time of data collection more female students were on ground and were willing to be part of the study and also more of them returned their completed questionnaire. The high number of respondents of both south-east and south-south origin is not surprising since the University is situated in the south-south part of the country. Also, the high number of people who are of the Christian faith is because Christianity is the main religion of the people in the region.

The result of this study showed that more than a quarter of the respondents either stay alone or live with boy/girl friend. This further confirms the assertion that young people often take advantage of freedom from direct parental supervision and guidance to express their freedom by initiating sexual activity without adequate protection [15]. Indeed, the environment in higher institutions of learning in Nigeria, like in many other parts of the world, is characterised by high level of personal freedom and social interactions. Socially, the typical university environment in Nigeria offers opportunities for high level of sexual networking, and the freedom that characterizes the higher institutions permits permissive lifestyle [16].

### Respondents risk behaviours

Many of the respondents had ever taken alcohol before, with more than a quarter been current users. This finding corroborates the findings of Maharaj, Nunes, and Renwick who found out that 24% of adolescents in English-speaking Caribbean, had used cigarettes and 17% marijuana [17]; this might be because adolescents substance abuse usually starts with alcohol and cigarette which are referred to as gateway substances [18]. The easy accessibility of young people in most of our communities to these substances might be responsible for this among the study population. The need to step up the call for actual ban of young people to these substances which have the ability to impede on their senses is very important and urgent.

The result of this study also shows that more than a quarter of the respondents have parents that drinks alcohol, this agrees with the study of Brook, Morojele, Pahl and Brook which asserts that young people whose parents and caregivers use alcohol and other drugs are more inclined than those who do not experience drug-taking in their homes to also use them. Adolescents who are exposed to such behaviour are more likely to model it and also consider it acceptable [19].

A relationship was established between current use of alcohol and sexual experience in this study. This finding calls for a spirited effort from concerned authority and stakeholders to urgently address the issue of alcohol intake among young persons. Studies have shown that young persons who drink alcohol and/or use other drugs are more likely to be sexually active than are those who do not, and also more likely to engage in unprotected sex [20, 21] and these are associated with having unplanned pregnancies [22] and contracting sexually transmitted infections, including HIV. The use of substances including alcohol is reported to decrease young persons’ inhibitions and safer sex negotiation skills, thereby increasing their already-present vulnerability to engaging in sexual risk behaviour [23].

### Respondents sexual behaviour

In Nigeria, several studies across the regions have shown that sexual activity among adolescents is high and increasing [2, 24, 25]. These studies also confirmed that premarital sex occurs and appears to be increasing as adolescents delay marriage for the purpose of acquiring formal education [2, 24, 25]. In this study, more than half of the respondents have either boy/girlfriend and a total of 144 (52.0%) of the respondents have ever had sex with someone before. This is revealing in that it shows that more young people in higher institutions are getting sexually active and most lack the necessary reproductive health information to practice safe sexual practices. This finding also agrees with the findings of Manning, Giordano and Longmore which claims that 75% of youth had casual sex with a friend, ex-girlfriend or boyfriend [26]. However, in a study in India, the result reveals a contrary view that students in tertiary institution first sexual experience was unplanned (65%), others reported that it was forced (14%). As far as overall experiences were concerned, partners were overwhelmingly a boyfriend or girlfriend, that is, a romantic partner and multiple and sexual encounters were rare [27]. The findings in this study further confirmed the assertion that sex is a phenomenon currently ravaging higher institution in Nigeria as a lot of students are engaged in premarital and heterosexual relationships on campus [28].

Respondents age at first sexual intercourse revealed that a little below half had their first sexual intercourse within the age range of 5-24 years. This call for attention of those in authority to have a fresh look into the issue of child sexual molestation since these under-aged might have been tricked into the act without their consent; the present situation where people turn their eyes away from this heinous act as if it is non-existence in the country will not help the victims because of the psychological, social and health consequences on them both in the present and in the future. This findings further corroborates the findings in the 2008 NDHS which revealed that initiation of sexual activity before marriage is not uncommon in Nigeria as respondents aged 25-49 years, the median age at first sexual intercourse is 17.7 years for women and 20.6 years for men and the 2003 NDHS survey which revealed that over 16% of teenage females had first sexual intercourse by age 15 and among young women ages 20 to 24, nearly half (49.4%) reported first sexual intercourse by age 18; among teenage males, 8.3% reported first sexual intercourse by age 15 and among those ages 20 to 24, 36.3% reported first sexual intercourse by age 18. [2].

The result showed that more than a quarter of the respondents had had sexual intercourse in 2011 when the study was conducted and some even had sex in exchange for gifts with a friend. This findings agrees with a recent study among students of the University of Ilorin who are within the same age range with those of the study population, 72.7% of the respondents had ever had sex, 62.3% had had more than one (1) sexual partner in the last 2 months preceding the study, with 30.0% of the respondents having between 2-3 partners, 25.9% had had between 4-6 partners, while 6.4% had had more than 6 partners within the same period. [29]. Also, the result confirmed the result of the 2008 NDHS which shows that only 11% of sexually active men and women age 15-19 ever use of a modern contraceptive method [2]. This call to question the various efforts from stakeholders to address the issue of risky behavior among young people most especially those in higher institutions in the recent past. With the high level of awareness of HIV/AIDS among young people in Nigeria as reported by Omoregie (2002) [30], Adedimeji (2003) [31], and the 2008 NDHS [2], one would have expected that this knowledge would have translated to practice but this result is pointing to the contrary. Risky sexual acts are still common occurrences among students in higher institution. For example, in a research conducted on fresh students of tertiary institutions in Rivers state, Ibe (2003) [32], observed the risky practices recorded among students to include having sex without condom (57.0%), having had multiple sexual partners (42.1%) and use of condom at first sexual encounter (22.8%). Some had multiple current partners with 3.5% having 4 to 6 current partners. Various factors have been adduced for these risky behaviors among young people in Nigeria, these include: lack of communication between parents and children about sex; high level of illicit sexual activity; high incidence of campus prostitution, poverty or hash economic conditions among other factors [33, 34]. The school authority needs to strategize to find a better way of using the high knowledge to change both behavior and practice if the war against STIs and HIV/AIDS will ever be won in Nigeria and among young people in particular.

## Conclusion

In conclusion, the findings of this study showed that risky sexual behavior exist among the respondents and few of the sexually active used any form of protection during their last sexual episode. There is the need to step up campaigns to address this noticed lapse in behavior among the students in order to arrest the usual consequences of such risky sexual behavior.

## References

[1] World Bank Reproductive Health Action Plan 2010–2015 (RHAP) http://www.worldbank.org/population.

[2] National Population Commission (NPC) [Nigeria] and ORC Macro (2008). Nigeria Demographic and Health Survey 2009.

[3] Singh. SA, Wulf D Early Child bearing in Nigeria: A Continuing Challenge.

[4] Moronkola OA, Idris OM (2000). Sexual health knowledge, determinants of sexual behaviour and use of contraceptives among female secondary school students in Ibadan, Nigeria. Niger School Health..

[5] Esiet A (2003). Building support for adolescent health education and services in Nigeria: reflections from the experience of Action Health Incorporated (AHI).

[6] Murray N, Winfrey W, Chatterji M, Moreland S, Dougherty L, Okonofua F (2006). Factors Related to Induced Abortion among Young Women in Edo State, Nigeria. Studies in Family Planning.

[7] Arowojolu AO, Ilesanmi AO, Roberts OA, Okunola MA (2002). Sexuality, contraceptive choice and AIDS awareness among Nigerian undergraduates. African Journal of Reproductive Health..

[8] Bankole A (2004). Risk and Protection: Youth and HIV/AIDS in Sub-Saharan Africa.

[9] Sedgh G, Bankole A, Okonofua F, Imarhiagbe C, Hussain R, Wulf D (2009). Meeting Young Women's Sexual and Reproductive Health Needs in Nigeria.

[10] Iwuagwu SC, Ajuwon AJ, Olasheha IO (2000). Sexual behaviour and negotiation of male condoms by female students of the University of Ibadan. Journal of Obstetrics and Gynaecology..

[11] Echendu DA, Joseph IBA, Nkemakolam OE, Chima I, Akinsewa A, Ejike O (2011). Awareness and use of contraception by women seeking termination of pregnancy in south eastern Nigeria. Asian Pacific Journal of Tropical Disease.

[12] Federal Ministry of Health. National HIV/AIDS Reproductive Health Survey (2007).

[13] UNAIDS (2006). http://www.unaids.org.

[14] Federal Ministry of Health (FMOH) (2003). National HIV/AIDS and Reproductive Health Survey.

[15] Iwuagwu SC, Ajuwon AJ, Olasheha IO (2000). Sexual behaviour and negotiation of male condoms by female students of the University of Ibadan. Journal of Obstetrics and Gynaecology..

[16] Fatusi AO, Akinrinade S, Kolawole M, Mojola I, Ogungbile Faith Communities and Adolescent Sexual Health Development in HIV/AIDS Era.

[17] Maharaj RG, Paula N, Shamin R (2009). Health risk behaviours among adolescents in the English-speaking Caribbean: a review. Child Adolesc Psychiatry Ment Health..

[18] McArdle P (2004). Substance use by children and young people. Arch Dis Child..

[19] Brook JS, Morojele NK, Pahl T, Brook D (2006). Predictors of drug use among South African Adolescents. Journal of Adolescent Health..

[20] Pluddemann A, Flisher A, Mathews C, Carney T, Lombard C (2008). Adolescent methamphetamine use and sexual risk behaviour in secondary school students in Cape Town, South Africa. Drug and Alcohol Review..

[21] Moshia KM, Brook JS, Morojele NK (2004). A comparison of risky sexual behaviours between adolescent substance users and non-substance users in South Africa.

[22] Vundule C, Maforah F, Jewkes R, Jordaan E (2001). Risk factors for teenage pregnancy among sexually active black adolescents in Cape Town: A case control study. South African Medical Journal..

[23] Morojele NK, Brook JS, Kachieng'a MA (2006). HIV/AIDS, sexual risk behaviour and substance abuse among adolescents in South Africa: A qualitative investigation. AIDS Care..

[24] Bankole A, Oye-Adeniran BA, Singh S, Adewole IF, Wulf D, Sedgh G, Hussain R (2006). Unwanted Pregnancy and Induced Abortion in Nigeria: Causes and Consequences.

[25] Federal Ministry of Health (2005). National HIV/AIDS Reproductive Health Survey.

[26] Manning WD, Giordano PC, Longmore MA (2006). Hooking up: The relationship context of “nonrelationship” sex. Journal of Adolescent Research..

[27] Rachna S Premarital Sexual Behaviour among Unmarried College Students of Gujarat.

[28] Magnus OO, Gbakeji JO (2009). Analysis of Spatial Awareness of HIV/AIDS amongst students of Tretuary Institutions in Edo State, Nigeria. Journal of Ethno- medicine..

[29] Fawole AO, Ogunkan DV, Adegoke GS (2011). Sexual Behaviour and Perception of HIV/AIDS in Nigerian Tertiary Institutions: University of Ilorin, a Case Study. Global Journal of Human Social Science.

[30] Omoregie GO (2002). Sexual behaviour of tertiary institution students using the PSI behavioural change framework.

[31] Adedimeji A (2003). Perception of HIV/AIDS infection and condom use among undergraduates in Nigerian universities.

[32] Ibe SN, Ibe AU (2003). Condon use among sexually active students in Bori, Rivers State, Nigeria. African Journal of Applied sciences and Environmental Biology..

[33] Uzokwe AO Prostitution in Nigerian University Campuses (part1) Nigerian World (Monday, July, 21. 2008). http://htpp//www.nigeriaworld.com.

[34] Obinna C Story that Touches the Heart: Why Prostitution rate is rising.

